# PCBP1 interacts with the HTLV-1 Tax oncoprotein to potentiate NF-κB activation

**DOI:** 10.3389/fimmu.2024.1375168

**Published:** 2024-04-16

**Authors:** Rui Su, Xue Kang, Yifan Niu, Tiesuo Zhao, Hui Wang

**Affiliations:** ^1^ Department of Immunology, School of Basic Medical Sciences, Xinxiang Medical University, Xinxiang, China; ^2^ Henan Key Laboratory of Immunology and Targeted Drug, Xinxiang Medical University, Xinxiang, China; ^3^ Xinxiang Engineering Technology Research Center of Immune Checkpoint Drug for Liver-Intestinal Tumors, Xinxiang Medical University, Xinxiang, China; ^4^ Henan Collaborative Innovation Center of Molecular Diagnosis and Laboratory Medicine, School of Medical Technology, Xinxiang Medical University, Xinxiang, China

**Keywords:** PCBP1, HTLV-1 tax, IKK-NF-κB signaling, cell proliferation, interaction

## Abstract

Human T-cell leukemia virus type 1 (HTLV-1) is the etiological agent of adult T-cell leukemia/lymphoma. The HTLV-1 Tax constitutively activates nuclear factor-κB (NF-κB) to promote the survival and transformation of HTLV-1-infected T cells. Despite extensive study of Tax, how Tax interacts with host factors to regulate NF-κB activation and HTLV-1–driven cell proliferation is not entirely clear. Here, we showed that overexpression of Poly (rC)–binding protein 1 (PCBP1) promoted Tax-mediated IκB kinase (IKK)–NF-κB signaling activation, whereas knockdown of PCBP1 attenuated Tax-dependent IKK–NF-κB activation. However, Tax activation of HTLV-1 long terminal repeat was unaffected by PCBP1. Furthermore, depletion of PCBP1 led to apoptosis and reduced proliferation of HTLV-1–transformed cells. Mechanistically, PCBP1 interacted and co-localized with Tax in the cytoplasm, and PCBP1 KH3 domain was indispensable for the interaction between PCBP1 and Tax. Moreover, PCBP1 facilitated the assembly of Tax/IKK complex. Collectively, our results demonstrated that PCBP1 may exert an essential effect in Tax/IKK complex combination and subsequent NF-κB activation, which provides a novel insight into the pathogenetic mechanisms of HTLV-1.

## 1 Introduction

Human T-cell leukemia virus type 1 (HTLV-1), the first identified human retrovirus in the 1980s (1), is associated with the development of adult T-cell leukemia/lymphoma (ATL), a highly aggressive malignancy of CD4^+^ T lymphocytes. HTLV-1 infection can also cause neurological disorder termed HTLV-1–associated myelopathy/tropical spastic paraparesis (HAM/TSP) (2). It has been reported that HTLV-1 infects more than 20 million people worldwide, although most of them remain asymptomatic throughout their lifetime, approximately 5% of carriers develop into ATL or HAM/TSP after many years of latency (3, 4). The endemic regions of HTLV-1 are distributed in South America, southern Japan, the Caribbean, equatorial African, and central Australia (5).

HTLV-1 encodes a 40-kDa transactivator/oncoprotein, Tax, which acts an important role in regulating viral gene transcription (6). Tax activates HTLV-1 gene expression through recruiting cyclic adenosine monophosphate (cAMP) response element–binding protein/activating transcription factor (CREB/ATF) and coactivators CBP/p300 to the HTLV-1 long terminal repeat (LTR) promoter (7, 8). In addition, Tax confers malignant transforming capacity to HTLV-1 (9). The expression of Tax can induce the immortalization of primary human CD4^+^ lymphocytes and the transformation of murine fibroblasts (10, 11), and the transgenic mice of Tax develop lymphoma and leukemia (12, 13). Tax is involved in HTLV-1–induced proliferation and malignant transformation by modulating a variety of signaling pathways including the activation of nuclear factor-κB (NF-κB), phosphatidylinositol 3-kinase-protein kinase B (PI3K-AKT), serum response factor, and yes-associated protein (14–16), as well as inhibiting tumor suppressor Rb, p53, and DLG1 (17, 18).

Tax persistently activates canonical and noncanonical NF-κB pathways, which drives inflammation and malignant transformation of HTLV-1–infected T cells (14, 19). The activation of canonical NF-κB pathway by Tax begins with the phosphorylation of IκB kinase (IKK) complex, which is composed of one regulatory subunit, NEMO (also known as IKKγ), and two catalytic subunits, IKKα and IKKβ (20). Through direct interaction with NEMO, Tax induces the oligomerization of IKK complex in a K63/Met1-linked hybrid polyubiquitin chain–dependent manner and modulates downstream signaling by mediating phosphorylation, ubiquitination, and subsequent proteasomal degradation of the inhibitor protein IκBα, thus releasing NF-κB for translocation into the nucleus and ultimately activating the transcription of NF-κB target genes (19, 21, 22). Tax activates the noncanonical NF-κB through the IKKα-dependent processing of p100 precursor protein to p52 subunit (23). The polyubiquitination modification of Tax is important for NF-κB activation. It has been shown that ubiquitin modification proteins such as E2 enzyme Ubc13, E3 ligase ring finger protein 4 (RNF4), TNF receptor associated factor 6 (TRAF6), ubiquitination factor E4B (UBE4B), and linear ubiquitin chain assembly complex (LUBAC) are tightly related to the process of Tax-dependent IKK–NF-κB activation (22, 24–27), whereas ubiquitin-specific peptidase 20 (USP20) suppresses Tax-induced NF-κB activation through deubiquitination of TRAF6 and Tax (28). Besides, several selective autophagy receptors including Tax1 binding protein 1 (TAX1BP1), NEMO-related protein (NRP), and sequestosome-1 (SQSTM-1/p62) also interact with Tax and regulate the Tax/IKK signalosome to potentiate Tax-dependent NF-κB activation (29–31). However, class II major histocompatibility complex transactivator (CIITA) suppresses Tax-mediated IKK activation by retaining Tax/p65 complex in the cytoplasm and blocking the transcriptional activity of p65 in nuclear level (32). Taken together, many host proteins exert essential functions in Tax-mediated IKK–NF-κB activation.

Poly (rC)–binding protein 1 (PCBP1) is a member of heterogeneous nuclear ribonucleoprotein (hnRNP) family and is closely associated with the transcriptional and post-transcriptional regulation (33). PCBP1 not only can bind to promoters of multiple genes to regulate gene transcription but also plays an important function in various physiological activities such as stabilizing mRNA and regulating alternative splicing of precursor mRNA (34, 35). In viral infection, PCBP1 enhances the replication levels of some virus by binding to its 5′-untranslated region (5′-UTR) including Enterovirus D68 (EVD68) and Enterovirus 71 (EV71) (36, 37) and mediates the degradation of mitochondrial antiviral signaling protein to inhibit RIG-I–like receptors signaling (38). Besides, PCBP1 facilitates the binding of cyclic GMP-AMP synthase (cGAS) and viral DNA to positively regulate the innate immune response (39). Recent studies showed that PCBP1 is a potential tumor suppressor and is related to the occurrence, maintenance, and metastasis of multiple types of tumors, including breast cancer, gastric cancer, hepatocellular carcinoma, and acute myeloid leukemia (40, 41). However, there have been no studies on the relationship between PCBP1 and HTLV-1 to date, which presents a major obstacle for understanding the function and molecular mechanisms of PCBP1 in HTLV-1 infection.

In this study, we assessed the effect of PCBP1 on Tax activation of NF-κB. We found that PCBP1 contributes to Tax- and HTLV-1–driven IKK–NF-κB signaling activation. Further studies revealed that PCBP1 interacts with Tax, and PCBP1 enhances the association of Tax and IKK complex. Thus, we identified a new host regulator of Tax and illustrated a novel mechanism underlying the regulation of IKK–NF-κB pathway during HTLV-1 infection.

## 2 Materials and methods

### 2.1 Cell cultures

Human embryonic kidney cell line (HEK293T) and a human cervical cancer cell line (HeLa) were obtained from American Type Culture Collection (Manassas, VA, USA) and maintained in Dulbecco’s modified Eagle’s medium (Gibco, Grand Island, NY, USA), supplemented with 10% fetal bovine serum (FBS) (Gibco) and 1% penicillin-streptomycin. HTLV-1–transformed cell lines MT2 and MT4 were described previously (42). Jurkat, MT2, and MT4 cells were grown in RPMI 1640 medium containing 1% penicillin-streptomycin and supplemented with 10% FBS. All cells were cultured at 37°C in a 5% CO_2_ incubator.

To generate stable Jurkat-shNC, Jurkat-shPCBP1, MT2-shNC, MT2-shPCBP1, MT4-shNC, MT4-shPCBP1, HEK293T-shNC, and HEK293T-shPCBP1 cell lines, HEK293T cells were seeded into 6-well plate and transfected with 2 μg of PLKO.1 vector encoding shNC or shPCBP1, 1.5 μg of psPAX2, and 0.5 μg of pMD2.G plasmids with Lipofectamine 2000 (Lipo2000) (Invitrogen, Carlsbad, CA, USA) according to the manufacturer’s instructions. At 24 h and 48 h after transfection, the lentivirus was collected from HEK293T supernatants. After incubation with the lentivirus for 48 h, the Jurkat, MT2, MT4, and HEK293T cells were selected by puromycin (2.5 μg/mL; Sigma-Aldrich, St. Louis, MO, USA), and the knockdown efficiencies of shRNAs were determined by Western blotting.

### 2.2 HTLV-1 infection

HeLa cells (1 × 10^6^) were seeded into 6-well plate and cocultured with MT2 cells at the ratio of 1:1 or 1:2. After indicated time, the mixed cells were washed and shook several times with phosphate buffered saline (PBS) to remove MT2 cells. The remaining HeLa cells were collected for further analysis. For flow-cytometric analysis of HTLV-1–infected HeLa cells, the forward scatter (FSC)/side scatter (SSC) was acquired on a FACSCanto flow cytometer (BD Biosciences, CA, USA), and data were analyzed with FlowJo software (Tree Star, Inc., Ashland, OR).

### 2.3 Antibodies and reagents

Rabbit anti-hemagglutinin (HA) (H6908) and mouse anti-FLAG (F1804) antibodies were obtained from Sigma-Aldrich. Rabbit anti–phospho-IKKα/β (2697), anti-IKKβ (8943), anti–phospho-NF-κB p65 (3033), anti–NF-κB p65 (8242), anti–phospho-IκBα (2859), anti-IκBα (4812), anti-poly-ADP ribose polymerase (PARP) (9532), anti–cleaved-caspase3 (9661), Rat anti-CD3ϵ (4443), and mouse anti-IKKα (11930) antibodies were purchased from Cell Signaling Technology (Beverly, MA, USA). Mouse anti–HTLV-1 Tax (sc-57872) (ab26997) and anti-HTLV-1 p19 (ab9080) antibodies were obtained from Santa Cruz Biotechnology (Santa Cruz, CA, USA) and Abcam (Cambridge, United Kingdom), separately. Mouse anti-glyceraldehyde-3-phosphate dehydrogenase (GAPDH) (60004-1-Ig), β-actin (66009-1-Ig), anti-Myc (60003-2-Ig), anti-green fluorescent protein (GFP) (66002-1-Ig) antibodies, and rabbit anti-NEMO (18474-1-AP) and anti-CD4 (11056-2-AP) antibodies were purchased from Proteintech Group (Wuhan, China). Rabbit anti-PCBP1 (A1044) antibody was purchased from ABclonal Technology (Wuhan, China).

### 2.4 Plasmids and constructions

The PCBP1 gene was amplified from HeLa cells and the cDNA was inserted into pcDNA3.1 and pEGFP-C1 vector. The HTLV-1 Tax gene was obtained as described previously (43). The DNA fragments of Tax were ligated into pCAGGS-HA and pCMV-Myc vector to generate plasmids expressing HA or Myc-tagged Tax.

The short-hairpin RNAs (shRNAs) were clone into PLKO.1 vector. The target sequences for the negative-control and human PCBP1 were designed as follows: shNC, 5′-CAACAAGATGAAGAGCACCAA-3′; shPCBP1-2, 5′-AAGGCTTTCGCTATGATCATC-3′. Small interfering RNA (siRNA) oligonucleotides target for negative-control and human PCBP1 were designed and synthesized by GenePharma (Shanghai, China). The siRNA (50 nM) was transfected into HeLa cells with Lipo2000. At 24 h post-transfection of siRNA, the cells were transfected with indicated plasmids. The sequences of all siRNA are as follows: siNC, 5′-UUCUCCGAACGUGUCACGUTT-3′, siPCBP1, 5′-GGGAGAGUCAUGACCAUUCTT-3′.

### 2.5 Dual-luciferase reporter assays

HeLa cells were plated on 24-well plate and transiently transfected with 10 ng of pRL-TK *Renilla* luciferase reporter plasmid, 100 ng of NF-κB firefly luciferase reporter plasmid and indicated expression plasmids. After 24 h of DNA transfection, the luciferase reporter activities were determined by a dual-specific luciferase assay kit (Promega, Madison, WI, USA) for analysis with a luminometer (Promega). Firefly luciferase activities were normalized on the basis of *Renilla* luciferase activities. The results were represented as relative luciferase activity.

### 2.6 Western blotting and co-immunoprecipitation

The cells were plated in 6-well plate, and transient transfection was implemented by mixing with plasmids and transfection reagent Lipo2000. After DNA transfection for 24 h, the cells were lysed in 1 mL of radio immunoprecipitation assay (RIPA) lysis buffer [50 mM Tris-HCl, 150 mM NaCl, 0.25% sodium deoxycholate, and 1% NP-40 (pH 7.4)] supplemented with protease inhibitor cocktail (Roche, Basel, Switzerland). For phosphoprotein detection, phosphatase inhibitor (Roche) was also added. The lysates were centrifugated at 12,000 rpm for 10 min in a 4°C centrifuge. A part of lysate was removed as input sample, and the rest of the supernatant was rotated with specific primary antibody overnight at 4°C. After that, the mixture was incubated with Protein G agarose (Santa Cruz) for another 2 h to collect the IP complex. After washing four times with 1 mL of RIPA buffer, the pellets were resuspended and denatured with sodium dodecyl-sulfate polyacrylamide gel electrophoresis (SDS-PAGE) loading buffer, loaded onto 10% SDS-PAGE gel. The separated proteins were transferred onto a polyvinylidene difluoride membrane (Millipore, Burlington, MA, USA). The membranes were blocked with 5% skim milk in PBST (PBS with 0.1% Tween) for 2 h at room temperature and incubated with the primary antibodies at 1:1,000 dilution overnight at 4°C. After being washed three times with PBST, the membranes were incubated with peroxidase-conjugated anti-rabbit or anti-mouse IgG at 1:5,000 dilution for 1 h at room temperature. After washing three times with PBST, the band was detected with a luminescent image analyzer (Amersham Imager 600, GE, MA, USA).

### 2.7 Cell proliferation measure

Cell proliferation was measured with a cell counting kit 8 (CCK8) assay according to the manufacturer’s instructions (Kemix, China). In brief, Jurkat, MT2 or MT4 cells with 5,000 cells per well were seeded in 96-well plate in 100 μL of culture medium. At indicated times, 10 μL of CCK8 solution was added to each well for 1 h at 37°C in an incubator. The cell proliferation was determined by measurement the absorbance at 450 nm.

### 2.8 Confocal microscopy

The confocal microscopy was performed according to the method previously reported (44). Briefly, the cells were cultured on 20-mm coverslips and transfected with 0.5 μg of indicated plasmids for 24 h. After washing three times with PBS, the samples were fixed with 4% formaldehyde for 30 min, permeabilized using 0.4% Trixon X-100 for 5 min and incubated with 5% bovine serum albumin (BSA) for 30 min at room temperature. Then, the cells were probed with specific primary antibodies overnight at 4°C and stained with fluorescein isothiocyanate (FITC)–conjugated goat anti-rabbit immunoglobulin G (IgG) or Cy3-conjugated goat anti-mouse IgG (Proteintech Group) for 1 h at room temperature. The nuclei were imaged with 4′,6-diamidino-2-phenylindole (DAPI) (Roche) for 10 min at room temperature. After the last wash with PBS, confocal laser scanning microscopy (Leica Microsystem, Wetzlar, Germany) was used to analyze the stained samples.

### 2.9 Statistical analysis

All experiments were reproducible and repeated at least three times. All data were presented as means ± SD unless stated otherwise. Statistical testing was performed using Prism 8 software (GraphPad Software Inc., San Diego, CA, USA) with unpaired Student’s *t*-test. A *P*-value < 0.05 was considered as statistically significant.

## 3 Results

### 3.1 PCBP1 promotes Tax-mediated activation of NF-κB activity

**Figure 1:**
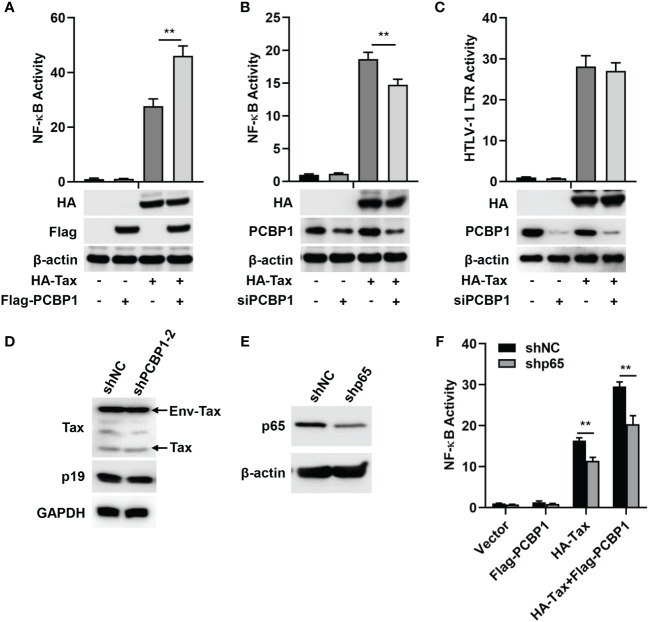
PCBP1 upregulates HTLV-1 Tax–mediated activation of NF-κB. **(A)** HeLa cells were transfected with pRL-TK, NF-κB luciferase, HA-Tax, and Flag-PCBP1 plasmids as indicated. NF-κB activity was measured by luciferase activity assays. The protein expression levels of HA-Tax, Flag-PCBP1, and β-actin were analyzed by Western blotting. **(B, C)** After transfection with siNC or siPCBP1 for 24 h, HeLa cells were transfected with pRL-TK, NF-κB, or HTLV-1 LTR luciferase along with Vector or HA-Tax expressing plasmids as indicated. The activities of NF-κB **(B)** and HTLV-1 LTR **(C)** were determined by luciferase activity assays. The protein levels of HA-Tax, PCBP1, and β-actin were examined by immunoblotting analysis. **(D)** MT2 stably expressing shNC and shPCBP1-2 cells were constructed and lysed. The proteins expression levels of Tax, p19, and GAPDH were analyzed by Western blotting. **(E)** HeLa cells were transfected with shNC or shP65 plasmids for 24 h, and the expression levels of p65 and β-actin in cell lysates were detected by Western blotting. **(F)** HeLa cells were transfected with shNC or shp65 for 24 h. Cells were then co-transfected with pRL-TK, NF-κB luciferase, HA-Tax, and Flag-PCBP1 expression plasmids as indicated. NF-κB activity was determined by luciferase activity assays. Data are shown as mean ± SD. **P < 0.01. Statistical significance was determined by Student’s *t-*test.

To determine the association between PCBP1 and HTLV-1, the effect of PCBP1 on Tax-regulated NF-κB or HTLV-1 LTR promoter activity was investigated. We initially showed that NF-κB activity was induced upon transfection of Tax plasmid in HeLa cells; moreover, such induction was enhanced by co-transfection of PCBP1 and Tax plasmids (**Figure 1A**). The role of PCBP1 in Tax-dependent activation of NF-κB and HTLV-1 LTR activity was further evaluated with a siRNA target PCBP1, which effectively attenuated PCBP1 expression in HeLa cells (**Figures 1B, C**). Our results indicated that the activity of NF-κB was upregulated by Tax in siNC expressing cells, whereas, in siPCBP1-expressing cells, the activity level of NF-κB was significantly reduced (**Figure 1B**), suggesting that PCBP1 exerts its function in NF-κB activity regulation upon the expression of HTLV-1 Tax. In contrast, Tax-induced HTLV-1 LTR activation was not affected after attenuating PCBP1 expression (**Figure 1C**), indicating that PCBP1 has no regulatory effect on HTLV-1 transcription. The protein expression levels of corresponding transfected plasmids were also confirmed by Western blot analysis (**Figures 1A–C**). At the same time, we applied lentiviruses expressing shRNA specific to PCBP1 with two independent sequences in HTLV-1–transformed cell lines, MT2. The expression of PCBP1 was reduced obviously by shPCBP1-2 (**Supplementary Figure 1**); thus, shPCBP1-2 was used for subsequent experiments. Compared with the MT2 cells expressing shNC, the viral Env-Tax, Tax, and p19 proteins were not affected in shPCBP1-treated MT2 cells (**Figure 1D**). Therefore, PCBP1 had no influence on HTLV-1 replication in HTLV-1–transformed cells.

The NF-κB pathway is persistently activated by Tax (14). As an one key component of NF-κB transcription factor family, p65 is translocated into nucleus to regulate target genes expression after stressful stimuli (45). With this in mind, we evaluated the role of p65 in PCBP1 and Tax-regulated NF-κB activity. Firstly, shRNA specific to p65 (shp65) was applied to impair the expression of p65 (**Figure 1E**). Subsequently, HeLa cells were treated with Tax expressing plasmid alone or Tax and PCBP1 expressing plasmids together in shNC and shp65 cells. We observed that NF-κB activity was increased by the sole expression of Tax protein or collective expression of Tax and PCBP1 proteins is shNC cells, whereas such increase was reduced in shp65 cells (**Figure 1F**). Taken together, our results showed that PCBP1 may selectively modulate Tax-induced NF-κB activation.

### 3.2 Overexpression of PCBP1 promotes the activation of IKK–NF-κB signaling by Tax protein and HTLV-1 infection

**Figure 2:**
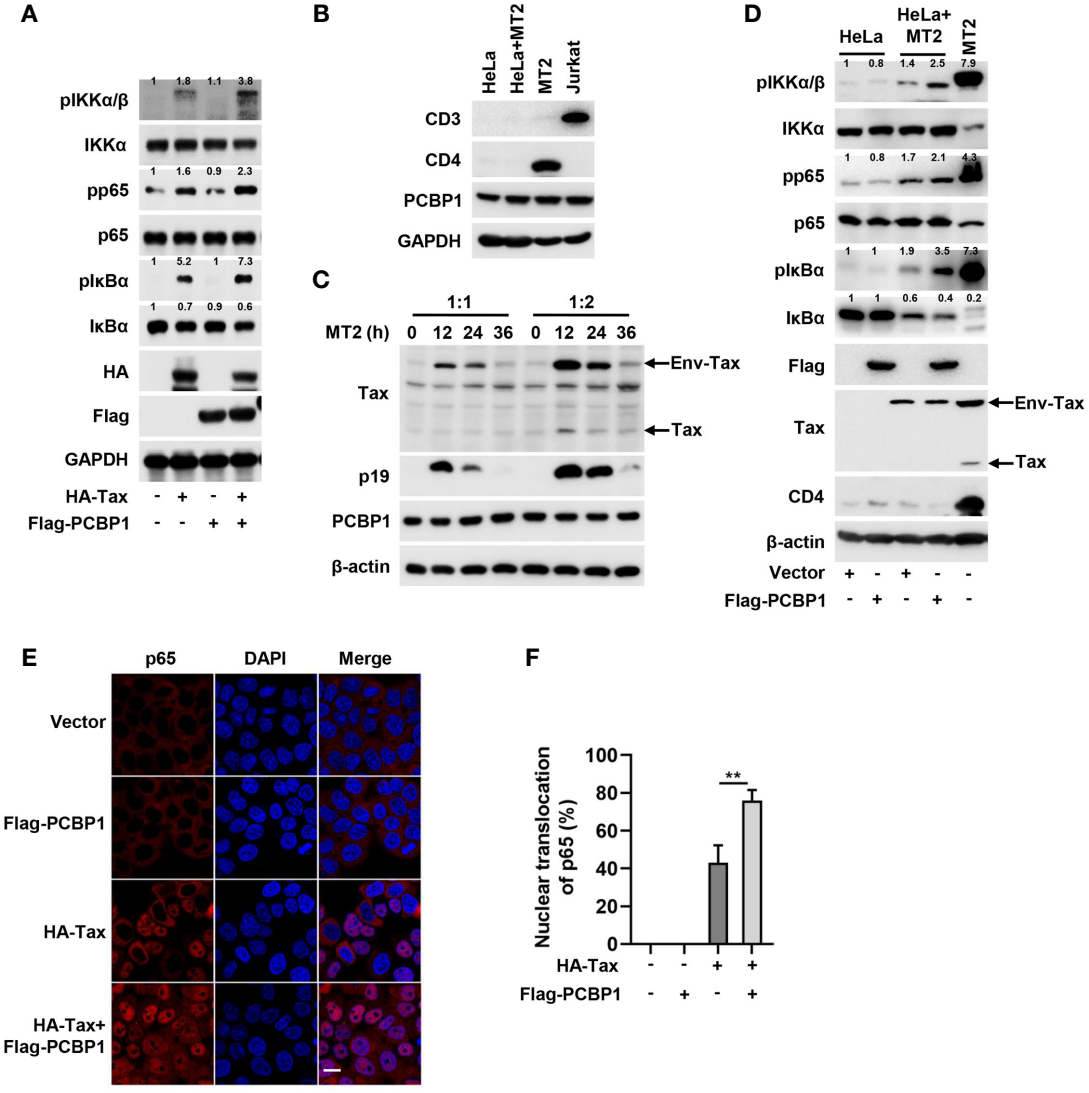
Overexpression of PCBP1 potentiates Tax-induced IKK–NF-κB signaling. **(A)** HeLa cells were transfected with HA-Tax, Flag-PCBP1, or HA-Tax and Flag-PCBP1 expression plasmids, respectively. The expression levels of various proteins in the cell lysates were detected by Western blot analyses and quantified with ImageJ software. **(B)** HeLa cells were cocultured with MT2 cells for 12 h; after that, the mixed cells were washed and shook with PBS several times to remove MT2 cells. The cell lysates of HeLa, HeLa after coculture with MT2, MT2 and Jurkat cells were prepared, and the levels of CD3, CD4, PCBP1, and GAPDH were determined by Western blotting. **(C)** HeLa cells were cocultured with MT2 cells at the ratio 1:1 or 1:2 for 0 h, 12 h, 24 h, and 36 h. Then, MT2 cells were removed, and the cell lysates were prepared for immunoblotting analysis of Tax, p19, PCBP1, and β-actin. **(D)** HeLa cells were transfected with Vector or Flag-PCBP1 plasmids alone for 24 h and then cocultured with MT2 cells for another 12 h; after that, the mixed cells were washed and shook with PBS several times to remove MT2 cells. The various proteins levels in the cell lysates were detected with corresponding antibodies and quantified with ImageJ software, and the MT2 cells were set as positive control. **(E, F)** HeLa cells were transfected with HA-Tax or Flag-PCBP1 expressing plasmid alone, or HA-Tax and Flag-PCBP1 expressing plasmids together as indicated. The localization of p65 was imaged as red and nucleus marker DAPI was indicated as blue by confocal microscopy **(E)**. Bar = 20 μm. The nuclear accumulation of p65 was quantified, and 40–50 cells from each genotype were analyzed **(F)**. All quantifications of images are representative of each group (n = 3) with similar results. Graphs show mean ± SD. **P < 0.01.

We explored the function of overexpressed PCBP1 on Tax-activated IKK–NF-κB signaling. Phosphorylation of IKKα/β, NF-κB p65, and IκBα was induced in cells transfected with Tax plasmid, and such inductions were further enhanced in cells co-transfected with Tax and PCBP1 plasmids and, at the same time, the endogenous expression of IκBα was further reduced in cells co-expressing PCBP1 and Tax compared with sole Tax transfected cells (**Figure 2A**), which indicates that PCBP1 regulates Tax-activated IKK–NF-κB signaling. Previous report revealed that cell-free HTLV-1 virions are hardly infectious for most cells, but cell-cell contact is more efficient for the transmission of the virus (46). Thus, the replication of HTLV-1 in HeLa cells was achieved through coculture of HeLa and MT2 cells. We first validated whether MT2 cells were completely removed after coculture by detecting T cell markers CD3 and CD4. As mentioned previously, MT2 cells did not express CD3 but expressed CD4 (47), and our results showed that the expression of CD4 was undetectable in HeLa after coculture with MT2 (**Figure 2B**). At the same time, we also compared the relative levels of endogenous PCBP1 in uninfected HeLa, HTLV-1–infected HeLa, MT2, and Jurkat cells, the results demonstrated that PCBP1 levels were similar in these cells (**Figure 2B**). Flow cytometry results indicated that there was no obvious MT2 population in HeLa after coculture (**Supplementary Figure 2**). Furthermore, the viral Env-Tax, Tax, and p19 proteins were clearly detected in HeLa cells after coculture, and the expression of PCBP1 was stable during HTLV-1 infection (**Figure 2C**). These results generally indicated that coculture model is effective to achieve HTLV-1 infection. Next, we evaluated the role of PCBP1 in HTLV-1–induced IKK–NF-κB activation. Upon HTLV-1 infection, the expression of pIKKα/β, pp65, and pIκBα increased and the expression of IκBα reduced, and such induction and reduction were enhanced in PCBP1 overexpressed cells (**Figure 2D**). Considering that the activation of IKK–NF-κB signaling leads to p65 translocation, the function of Tax and PCBP1 in nuclear accumulation of p65 was detected. Tax facilitated p65 translocation into nucleus; meanwhile, the co-transfection of PCBP1 and Tax further increased the nuclear accumulation of p65 (**Figures 2E, F**). Taken together, these results suggested that overexpression of PCBP1 significantly enhances Tax protein– and HTLV-1 infection–mediated IKK–NF-κB signaling activation, and PCBP1 functions on the upstream of IKK complex.

### 3.3 Knockdown of PCBP1 attenuates the activation of IKK–NF-κB signaling by Tax protein and HTLV-1 infection

**Figure 3:**
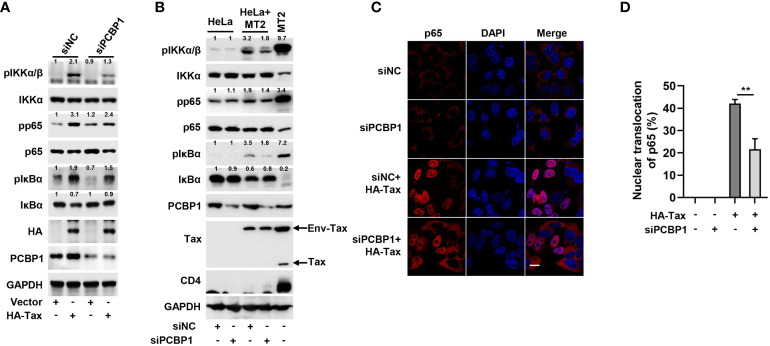
Knockdown of PCBP1 reduces Tax-induced IKK–NF-κB signaling. **(A)** HeLa cells were transfected with siNC or siPCBP1 for 24 h and then transfected with Vector or HA-Tax. The expression levels of various proteins in the cell lysates were detected by Western blot analyses and quantified with ImageJ software. **(B)** HeLa cells were transfected with siRNA target to negative-control or PCBP1 for 24 h, then cocultured with MT2 cells for another 12 h. Afterward, the cells were washed and shook with PBS several times to remove MT2 cells and lysed for immunoblot assays with corresponding antibodies, and the various proteins were quantified with ImageJ software. The sole MT2 cells were set as positive control. **(C, D)** HeLa cells were transfected with siRNA target to negative-control or PCBP1 and then transfected with Vector or HA-Tax plasmids as indicated. The localization of p65 was imaged as red and nucleus marker DAPI was indicated as blue by confocal microscopy **(C)**. Bar = 20 μm. The nuclear accumulation of p65 was quantified, 40-50 cells from each genotype were analyzed **(D)**. All quantifications of images are representative of each group (n = 3) with similar results. Graphs show mean ± SD. **, *P* < 0.01.

Further, we determined the association of PCBP1 with Tax-activated IKK–NF-κB signaling by knockdown of PCBP1. After transfection with siNC or siPCBP1, Tax plasmid was transfected into HeLa cells. In comparison to the negative control (siNC), the expression levels of phosphorylation of IKKα/β, NF-κB p65, and IκBα were upregulated by Tax transfection, but these productions were reduced in the presence of siPCBP1; the reduction of IκBα by Tax was recovered in cells expressing siPCBP1 rather than siNC (**Figure 3A**). Subsequently, we detected the effect of PCBP1 knockdown on HTLV-1–mediated IKK–NF-κB activation. The expression levels of pIKKα/β, pp65, and pIκBα were increased, and the expression of IκBα was reduced by HTLV-1 in cells expressing siNC, whereas such induction and reduction were attenuated by siPCBP1 (**Figure 3B**). Moreover, knockdown of PCBP1 significantly depressed Tax-mediated nuclear accumulation of p65 (**Figures 3C, D**). Altogether, the data indicated that PCBP1 plays an essential role in Tax protein– or HTLV-1 infection–mediated IKK–NF-κB signaling activation.

### 3.4 PCBP1 regulates IKK–NF-κB signaling and cell proliferation in HTLV-1–transformed cells

**Figure 4:**
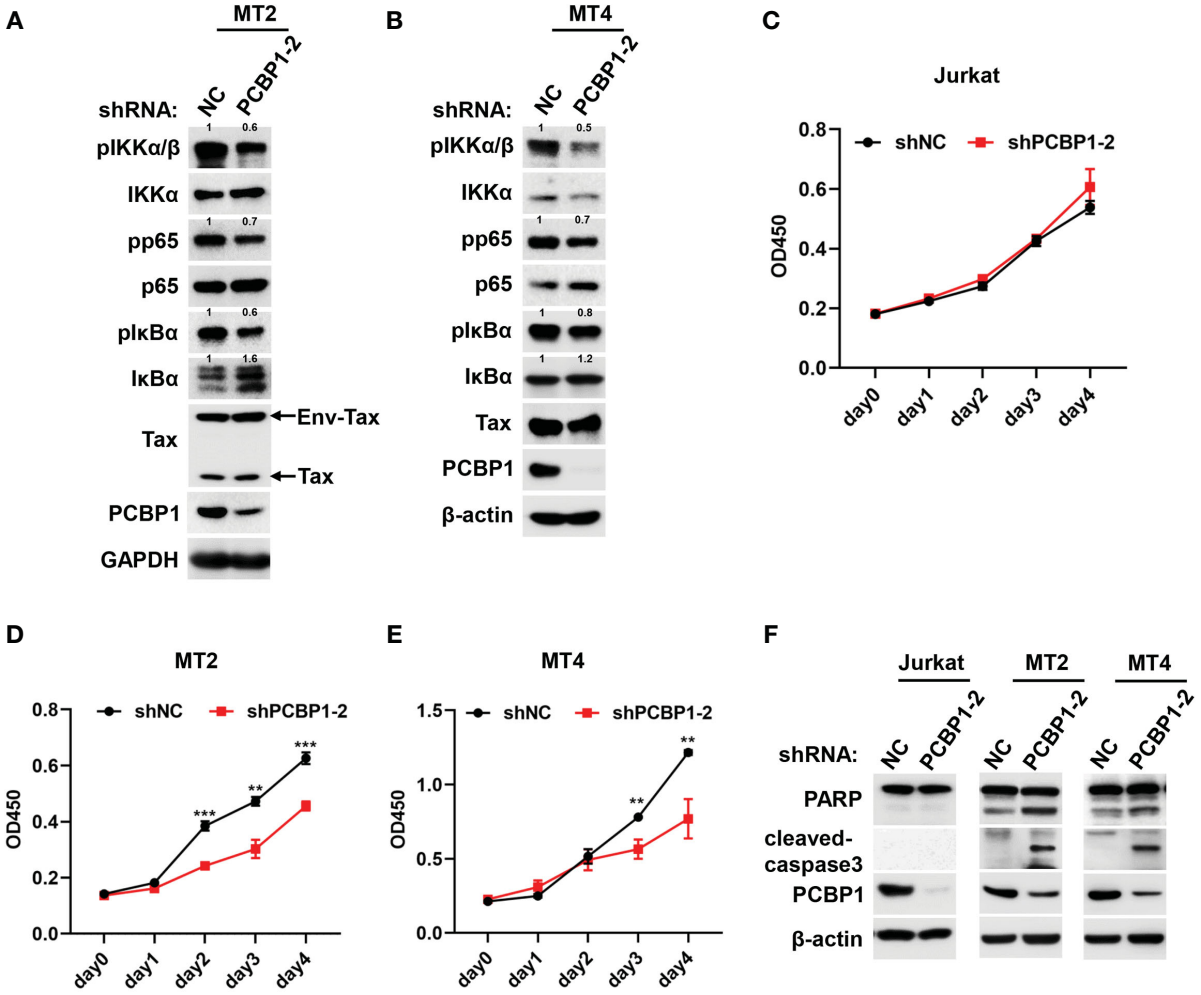
Knockdown of PCBP1 promotes apoptosis and inhibits proliferation in HTLV-1–transformed cells. **(A, B)** MT2 and MT4 stably expressing shNC and shPCBP1 cells were constructed and lysed. The levels of various proteins in cell lysates were analyzed by Western blotting, and the protein expression relative to internal control was quantified using ImageJ software. **(C–E)** Jurkat, MT2, or MT4 stably expressing shNC and shPCBP1 were seeded in 96-well plate with 5,000 cells per well, and the cell proliferation activity was determined by measurement the absorbance at indicated time at 450 nm, presenting as OD450. **(F)** Immunoblotting was performed with PARP, cleaved-caspase3, PCBP1, and β-actin antibodies using lysates from Jurkat, MT2, and MT4 cells stably expressing control shRNA or PCBP1 shRNA. Graphs show mean ± SD. **P < 0.01; ***P < 0.001.

In order to investigate whether PCBP1 supported Tax-mediated aberrant NF-κB activation in HTLV-1–transformed cells, except of MT2, we also applied lentiviruses to generate stably expressing shNC or shPCBP1 cell lines in another Tax expressing positive HTLV-1–transformed cell, MT4. Compared with the cells expressing shNC, the phosphorylation levels of IKKα/β, NF-κB p65, and IκBα were attenuated in shPCBP1-treated MT2 and MT4 cells (**Figures 4A, B**). Consistent with these findings, endogenous expression of IκBα was enhanced in shPCBP1 cells due to its impaired phosphorylation and proteasomal degradation (**Figures 4A, B**). Since NF-κB activation is important for HTLV-1–mediated oncogenesis and cell survival, the function of PCBP1 depletion on the viability and proliferation of HTLV-1–negative Jurkat cells and HTLV-1–transformed MT2 and MT4 cells was examined by CCK8 assay every 24 h, until day 4. Throughout the time course, the cells expressing shNC proliferated vigorously, whereas the cellular proliferation was reduced in shPCBP1-treated MT2 and MT4 cells, but not in control Jurkat cells (**Figures 4C–E**), demonstrating that PCBP1 promotes the proliferation of HTLV-1–transformed cells. We next explored the effect of PCBP1 knockdown on the apoptosis of Jurkat, MT2, and MT4 cells. In MT2 and MT4 shNC cells, the expressions of cleaved-PARP and cleaved-caspase3 were low; however, knockdown of PCBP1 enhanced the cleaved form of PARP and caspase3, but not in Jurkat cells (**Figure 4F**), which reveals that knockdown of PCBP1 induces the apoptosis of HTLV-1–transformed cell lines. Therefore, PCBP1 may regulate IKK–NF-κB signaling activation in HTLV-1–transformed cells, by which coordinates the cell proliferation and apoptosis in HTLV-1–transformed cells.

### 3.5 PCBP1 interacts with HTLV-1 Tax

**Figure 5:**
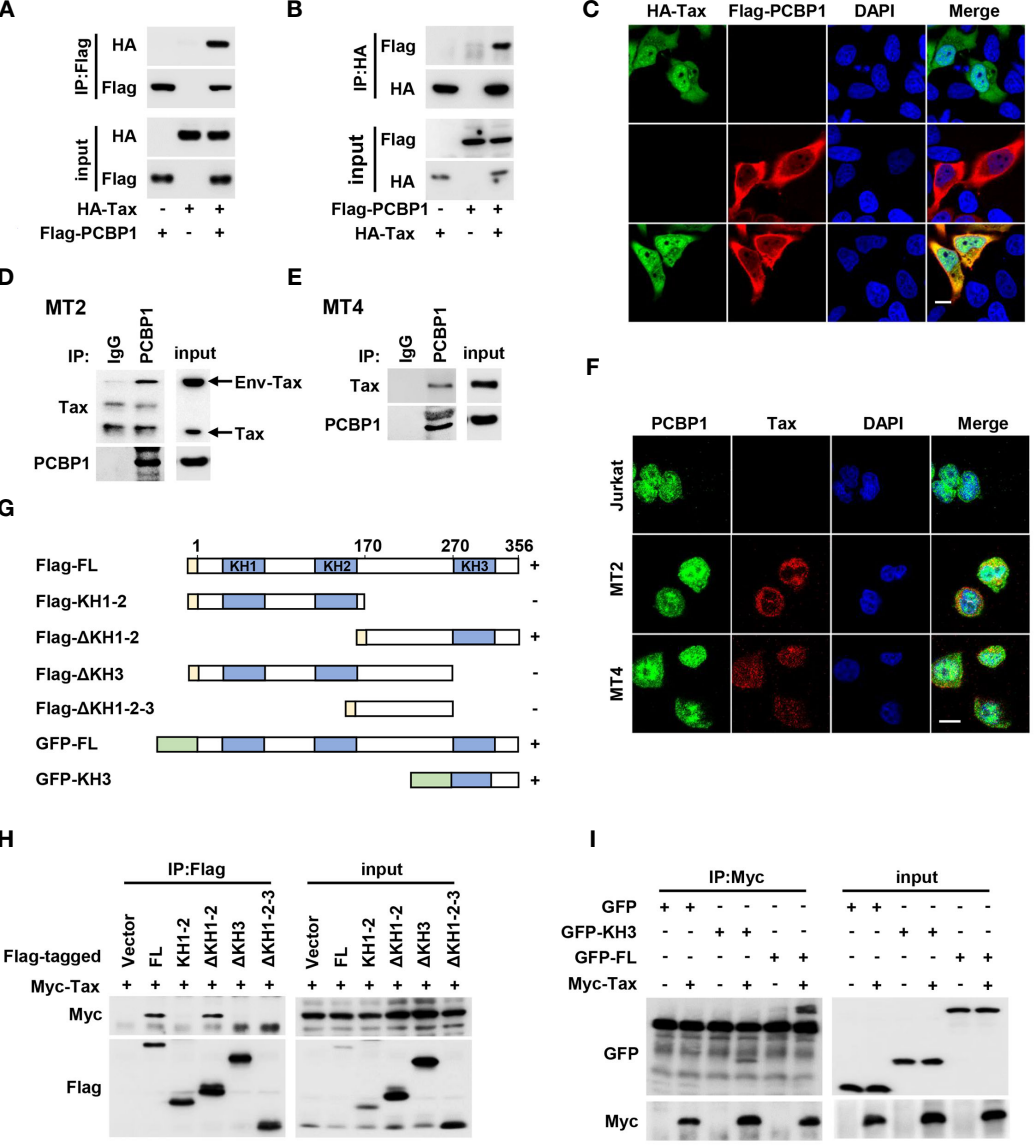
HTLV-1 Tax interacts with PCBP1. **(A, B)** HEK293T cells were plated in 6-well plate and transfected with the plasmids of HA-Tax and Flag-PCBP1 as indicated. Prepared whole-cell lysates were immunoprecipitated using Flag **(A)** or HA **(B)** antibody; the IP complex and input from whole-cell lysates were analyzed by immunoblotting analysis with HA and Flag antibody. **(C)** HeLa cells were transfected with HA-Tax and Flag-PCBP1. Cells were probed with HA (Green), Flag (Red) antibodies, and DAPI (Blue) before confocal microscopy analysis. Bar = 20 μm. **(D, E)** The cell lysates from MT2 **(D)** and MT4 **(E)** cells were immunoprecipitated with IgG or PCBP1 antibodies. The IP complex and whole-cell lysates were analyzed by Western blotting with antibodies to Tax and PCBP1. **(F)** Jurkat, MT2, or MT4 cells were probed with corresponding antibodies; the localization of PCBP1, Tax, and nucleus marker DAPI was presented as green, red, and blue, respectively. Bar = 20 μm. **(G)** The schematic for the construction of PCBP1 truncations. **(H)** After transfection together with Myc-Tax and Flag-PCBP1 FL or KH1-2, ΔKH1-2, ΔKH3, or ΔKH1-2-3 expressing plasmids for 24 h, the HEK293T cells were lysed and incubated with Flag antibody. The IP complex and whole-cell lysates were determined by Western blot analysis with antibodies to Myc and Flag. **(I)** HEK293T cells were transfected with plasmids as indicated for 24 h. Then, the cell lysates were immunoprecipitated with Myc antibody. The IP complex and whole-cell lysates were analyzed with the antibodies target to Myc and GFP. FL, full length.

To further understand the mechanism by which PCBP1 regulates Tax-mediated NF-κB activation, we performed a co-immunoprecipitation (Co-IP) experiment in HEK293T cells by co-transfected with plasmids expressing HA-Tax and Flag-PCBP1. We incubated the cell lysate with Flag antibody and Protein G for IP, and we detected HA-Tax in Co-IP and input samples when both proteins were overexpressed (**Figure 5A**). The cell lysate samples were also added with HA antibody and Protein G for IP, and Flag-PCBP1 was expressed in Co-IP and input samples when both proteins were expressed together (**Figure 5B**). Our reciprocal Co-IP results confirmed that PCBP1 interacted with Tax in overexpression conditions (**Figures 5A, B**). We also examined the interaction of PCBP1 and Tax inactive mutant, M22 and M47, which are deprived of NF-κB and CREB activation, respectively (24). Our results indicated that PCBP1 interacted with Tax M22 and M47, the same as Tax WT (**Supplementary Figure 3**). Previous studies demonstrated that Tax and PCBP1 are distributed in both cytoplasmic and nuclear compartments (37, 48). We co-transfected with HA-Tax and Flag-PCBP1 expressing plasmids in HeLa cells, and our confocal microscopy showed that HA-Tax and Flag-PCBP1 were mainly co-localized in cytoplasmic part (**Figure 5C**). Additionally, an endogenous Co-IP in HTLV-1–transformed cell lines MT2 and MT4 was performed. As expected, Env-Tax/Tax was detectable in anti–PCBP1-IP complex rather than anti-IgG-IP complex in both MT2 and MT4 cells (**Figures 5D, E**). The location of PCBP1 and Tax was also determined in HTLV-1–transformed cell lines. Immunofluorescence analysis showed that PCBP1 and Tax were distributed in both cytoplasmic and nuclear fraction, which is coincident with overexpressed results in HeLa cells, and PCBP1 was co-localized with Env-Tax/Tax in cytoplasm in MT2 and MT4 cells (**Figure 5F**). Jurkat cells were also immunostained as a negative control, and there was no Tax staining signal as expected (**Figure 5F**). Furthermore, we explored the interaction region of Tax and PCBP1. The different domain expressing plasmids were constructed (**Figure 5G**). Co-IP results revealed that Tax interacted with PCBP1 ΔKH1-2 domain, but not interacted with PCBP1 ΔKH1-2-3 domain (**Figure 5H**), implying that Tax may associate with PCBP1 KH3 domain. To verify such interaction, HEK293T cells were co-transfected with plasmids expressing Myc-Tax and GFP, GFP-PCBP1 KH3, or GFP-PCBP1 FL. The results further confirmed that Tax interacted with PCBP1 KH3 domain (**Figure 5I**). Taken together, these findings provided strong evidences of the interaction between Tax and PCBP1.

### 3.6 PCBP1 promotes the interaction of Tax and IKK complex

**Figure 6:**
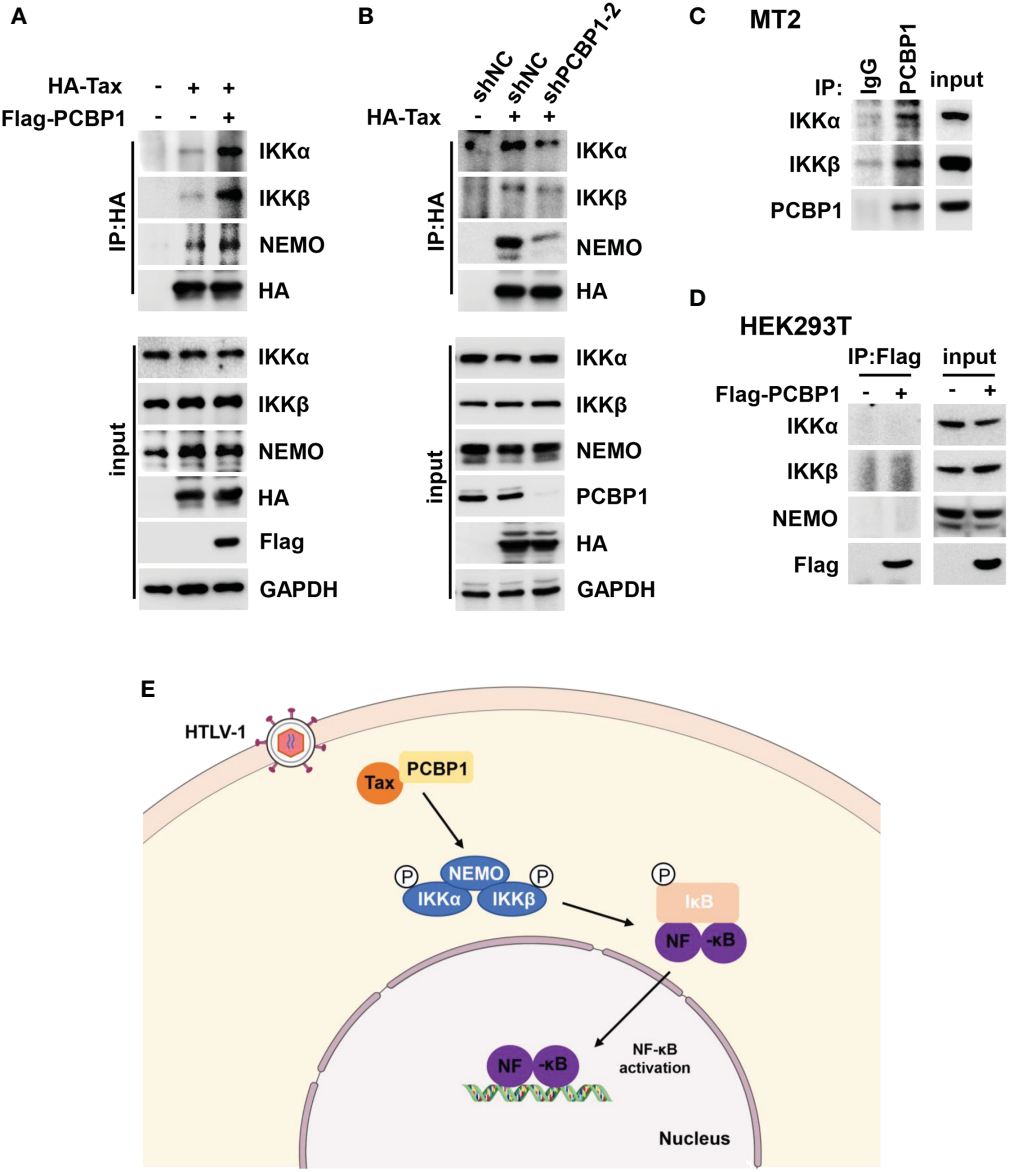
PCBP1 is important for the interaction between Tax and IKK complex. **(A)** HEK293T cells were transfected with the plasmids of HA-Tax and Flag-PCBP1 for 24 h. The cell lysates were immunoprecipitated with HA antibody and Protein G. The IP complex and whole-cell lysate were probed with IKKα, IKKβ, NEMO, HA, Flag, and GAPDH antibodies. **(B)** HEK293T stable expression shNC or shPCBP1 cells were generated and then were transfected with HA-Tax plasmids. The IP complex and whole-cell lysate were analyzed with IKKα, IKKβ, NEMO, PCBP1, HA, and GAPDH antibodies. **(C)** MT2 cells were lysed and immunoprecipitated with PCBP1 antibody and Protein G; the expression of IKKα, IKKβ, and PCBP1 in IP complex and input was determined by Western blotting. **(D)** HEK293T cells were transfected with Flag-PCBP1 plasmids. The cell lysates were immunoprecipitated with Flag antibody and Protein G. The IP complex and whole-cell lysate were probed with IKKα, IKKβ, NEMO, and Flag antibodies. **(E)** A hypothetical model underlying the function of PCBP1 in supporting Tax-mediated IKK–NF-κB signaling activation.

Because PCBP1 interacts with Tax and participates in Tax-mediated IKK–NF-κB signaling activation, the function of PCBP1 in Tax and IKK complex combination was investigated. It is previously reported that Tax can directly bind with NEMO, a subunit of IKK complex (49). Here, we detected Tax associated with the three subunits of IKK complex, IKKα, IKKβ, and NEMO, and such interactions were further increased by overexpression of PCBP1 (**Figure 6A**). Moreover, HEK293T cells stably expressing shNC or shPCBP1 were transfected with Tax expressing plasmid. The results indicated that knockdown of PCBP1 repressed the interaction between Tax and IKKα, IKKβ and NEMO (**Figure 6B**). At the same time, there was an interaction between PCBP1 and IKK complex in Tax-positive MT2 cells (**Figure 6C**), but not in Tax-negative HEK293T cells (**Figure 6D**). These results indicated that PCBP1 promotes the assembly of Tax and IKK complex. Taken together, these results revealed a mechanism by which PCBP1 interacts with HTLV-1 Tax to mediate NF-κB activation, leading to facilitating the proliferation of HTLV-1–infected cells (**Figure 6E**).

## 4 Discussion

The sustained activation of NF-κB pathways by HTLV-1 viral oncoprotein Tax initiates the inflammatory response and drives the development of ATL and HAM/TSP (9, 14, 49). In this study, we describe the role of PCBP1 in facilitating Tax-mediated NF-κB signal transduction and the proliferation of HTLV-1–transformed cells, which reveals a novel mechanism of Tax-activated IKK–NF-κB signaling and HTLV-1–mediated cell proliferation.

The association between hnRNP and HTLV-1 has been reported, such as hnRNP A1 interferes with the replication of HTLV-1 in T cells through inhibiting the binding of Rex to its response element (50, 51). Interestingly, our initial results showed that, as a hnRNP, PCBP1 enhanced the activation of Tax-mediated NF-κB luciferase activity but had no influence on the regulation of HTLV-1 LTR luciferase activity and HTLV-1 replication. These findings demonstrate that different members of hnRNP family perform different functions during HTLV-1 infection. Although previous study reported that PCBP1 is involved in the STAT3-mediated suppression of NF-κB activity (52). We discovered that PCBP1 was required for Tax protein– and HTLV-1 infection–induced activation of IKK–NF-κB signaling. PCBP1 promoted the phosphorylation of IKKα/β, p65, IκBα, and subsequent endogenous IκBα degradation, as well as the translocation of p65 into nucleus. Previous studies have shown that HTLV-1 Tax enhances the transcription of NF-κB target genes and inflammation response, thus driving HTLV-1–infected cells proliferation and immortalization, leading to ATL ultimately (14, 53). Here, we revealed that knockdown of PCBP1 diminished IKK–NF-κB signaling and cell proliferation, and triggered apoptotic cell death in HTLV-1–transformed cell lines, including MT2 and MT4. Therefore, the effect of PCBP1 in HTLV-1–transformed cells appears to be mainly through the regulation of IKK–NF-κB signaling.

Tax dynamically shuttles between the cytoplasm and nucleus for NF-κB activation and viral gene expression (54, 55). In cytoplasm, Tax directly interacts with NEMO within the cis-Golgi to persistently activate IKK–NF-κB signaling (56, 57). While in nucleus, Tax may regulate transcription, DNA damage response, and splicing (48, 55, 58). We showed that PCBP1 interacted with Tax and our confocal microscopy results further demonstrated that PCBP1 and Tax were mainly co-localized in cytoplasm, which is consistent with the location of Tax and IKK complex interaction, hinting the association between PCBP1 and Tax together with IKK complex. In fact, the confocal microscopy results also suggested that PCBP1 and Tax were partly co-localized in the nucleus. Previous reports have demonstrated that Tax dysregulates the cellular mRNA splicing in nucleus by interacting with some splicing factors including U2AF2 and DDX17 (58, 59). In addition, specific subtypes of some genes are involved in constant activation of the NF-κB pathway. The splicing variant of MyD88 in B-cell lymphoma maintains the activation of NF-κB pathway (60). Abnormal splicing of the tumor suppressor CYLD activates the NF-κB pathway, thereby boosting the development of chronic lymphoid leukemia (61). As an alternative splicing factor, PCBP1 is likely to be involved in Tax-reprogramed specific splicing events relevant to NF-κB activation. Therefore, how PCBP1 regulates alternative splicing and NF-κB activity in the context of Tax expression deserves further exploration.

We further revealed that overexpression of PCBP1 enhanced the interaction of Tax and IKK complex. In contrast, knockdown of PCBP1 attenuated the association of Tax and IKK complex. These findings indicated that PCBP1 may form a complex with Tax, regulatory subunit NEMO, and two catalytic subunits IKKα and IKKβ of IKK complex, which heightens the enzymatic activity of IKK and the following signaling pathway. Recent studies reported that Tax directly hijacks and aberrantly activates ubiquitin E3 ligase RNF8 and E2 conjugating enzymes Ubc13, to assemble K63-linked polyUb chains for IKK activation (62). Another study suggested that LUBAC promotes the activation of IKK complex by generating Met1-linked polyUb chains of Tax (22). Moreover, Tax also can be a ubiquitin E3 ligase to catalyzes the synthesis of free mixed-linked polyUb chains for the assembly of IKK complex and direct IKK activation (63). In addition, many Tax-binding proteins are critical for the activation IKK–NF-κB by Tax, including HSP90, p62, CDAM1, and TAX1BP1 (30, 31, 64). Based on this, we speculate that PCBP1-Tax interaction may function directly or influence the upstream mechanisms to govern IKK activity indirectly.

Indeed, there were some limitations in our study and several issues should be considered. Firstly, HeLa cells were selected to implement HTLV-1 infection experiment with some disadvantages, including HeLa naturally less likely to be infected by HTLV-1 and with a relatively high basal activity of NF-κB. Secondly, although our results preliminary suggested that knockdown of PCBP1 may impede the proliferation of HTLV-1–transformed cells, these findings should be further confirmed in ATL cells and ATL clinical samples. Thirdly, the specific mechanism of how PCBP1 regulates Tax/IKK complex assembly to enhance NF-κB activation has not been clearly explained. Even so, we verified that PCBP1 is involved in Tax-mediated NF-κB activation and the proliferation of HTLV-1–transformed cells.

In sum, the present study identified that PCBP1 as a novel Tax-interacting protein is recruited to the Tax/IKK complex to regulate Tax-mediated NF-κB activation. We demonstrated that PCBP1 plays an essential role in promoting proliferation and inhibiting apoptotic cell death of HTLV-1–transformed cells. Therefore, PCBP1 may represent an important regulatory mechanism of HTLV-1 Tax–mediated NF-κB activation and cell survival.

## Data availability statement

The original contributions presented in the study are included in the article/**Supplementary Material**. Further inquiries can be directed to the corresponding author.

## Ethics statement

Ethical approval was not required for the studies on humans in accordance with the local legislation and institutional requirements because only commercially available established cell lines were used.

## Author contributions

RS: Conceptualization, Data curation, Formal analysis, Funding acquisition, Investigation, Methodology, Validation, Visualization, Writing – original draft. XK: Data curation, Formal analysis, Investigation, Writing – original draft. YN: Data curation, Formal analysis, Investigation, Writing – original draft. TZ: Methodology, Supervision, Validation, Visualization, Writing – review & editing. HW: Conceptualization, Project administration, Resources, Supervision, Validation, Writing – review & editing.
