# Supplementary material for: PCBP1 interacts with the HTLV-1 Tax oncoprotein to potentiate NF-κB activation
Source: Front Immunol. 2024 Apr 16;15:1375168. doi: 10.3389/fimmu.2024.1375168 (PMC11058652; doi:10.3389/fimmu.2024.1375168)
Supplement: Supplementary file 1 [file DataSheet_1.docx]

**Supplementary Material**

**Supplementary Figure 1**


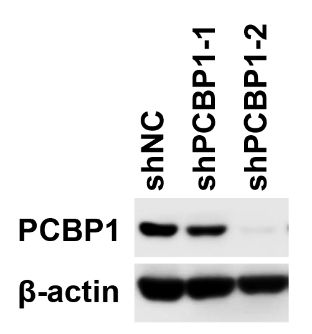


**Supplementary Figure 1. The knockdown effect of shRNAs specific to PCBP1.** The efficiencies of shRNAs target to PCBP1 in MT2 cells were evaluated by Western blotting with PCBP1 and β-actin antibodies.

**Supplementary Figure 2**


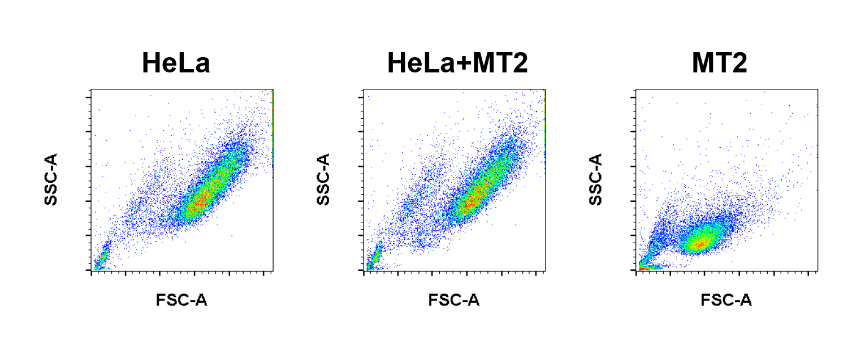


**Supplementary Figure 2. MT2 cells are removed after coculture with HeLa cells.** HeLa cells were cocultured with MT2 cells for 12 h, afterwards, the cells were washed and shook with PBS several times to remove MT2 cells. The remaining HeLa cells were detected with flow cytometry, and sole HeLa cells and MT2 cells without coculture were set as control.

**Supplementary Figure 3**


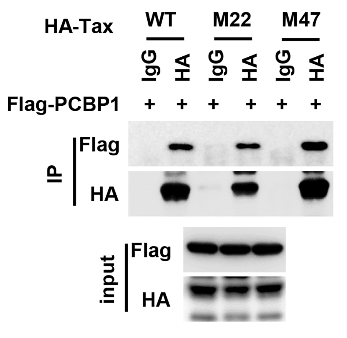


**Supplementary Figure 3. PCBP1 interacts with Tax mutants M22 and M47.** 293T cells were co-transfected with Flag-PCBP1 and HA-Tax WT, HA-Tax M22 or HA-Tax M47 plasmids in 6-well plate, respectively. The cell lysates were prepared and immunoprecipitated with IgG or HA antibody. The IP complex and input from whole-cell lysates were analyzed by Western blotting with HA and Flag antibodies.
